# ECG Multilead *QT* Interval Estimation Using Support Vector Machines

**DOI:** 10.1155/2019/6371871

**Published:** 2019-04-15

**Authors:** Jhosmary Cuadros, Nelson Dugarte, Sara Wong, Pablo Vanegas, Villie Morocho, Rubén Medina

**Affiliations:** ^1^Department of Electronics Engineering, Universidad Técnica Federico Santa Maria, Valparaiso, Chile; ^2^Research Center for Biomedical Engineering and Telemedicine, Electrical Engineering Department, Universidad de Los Andes, Mérida, Venezuela; ^3^Computer Science Department, Engineering School, Universidad de Cuenca, Cuenca, Ecuador

## Abstract

This work reports a multilead *QT* interval measurement algorithm for a high-resolution digital electrocardiograph. The software enables off-line ECG processing including *QRS* detection as well as an accurate multilead *QT* interval detection algorithm using support vector machines (SVMs). Two fiducial points (*Q*_ini_ and *T*_end_) are estimated using the SVM algorithm on each incoming beat. This enables segmentation of the current beat for obtaining the *P*, *QRS*, and *T* waves. The *QT* interval is estimated by updating the *QT* interval on each lead, considering shifting techniques with respect to a valid beat template. The validation of the *QT* interval measurement algorithm is attained using the Physionet PTB diagnostic ECG database showing a percent error of 2.60 ± 2.25 msec with respect to the database annotations. The usefulness of this software tool is also tested by considering the analysis of the ECG signals for a group of 60 patients acquired using our digital electrocardiograph. In this case, the validation is performed by comparing the estimated *QT* interval with respect to the estimation obtained using the Cardiosoft software providing a percent error of 2.49 ± 1.99 msec.

## 1. Introduction

Cardiovascular diseases are the leading cause of death globally, and they are also the first motivation for medical consultation. This health problem increases the need for medical service and has an important impact on health costs [1]. Among the different cardiovascular diseases, the arterial hypertension (HTA) [2], the coronary artery disease (CAD) [3], and the ischemic cardiomyopathy [4] are the most common. In particular, Chagas heart disease [5] is a parasite disease caused by the protozoan *Trypanosoma cruzi* leading to a progressive cardiac damage such as cardiomyopathy [6]. The disease is characterized by heart failure disease, arrhythmias that may predispose to systemic thromboembolism or sudden death, and atrioventricular block. The most frequent electrocardiographic abnormalities in patients with Chagas disease are ventricular extrasystole, complete block of right branch of His bundle, atrioventricular block, and ventricular fibrillation [7]. Chagas disease is also related to other cardiovascular diseases such as arterial hypertension and coronary artery disease [8]. However, the abnormalities vary depending on geographical region and status of the disease in each patient. The distribution of Chagas has changed in recent years due to permanent migrations [9].

In Chagas disease, one of the alterations that affect the electrocardiogram (ECG) signal is the abnormal variation of intervals such as the *QT* interval and morphology of the *T* wave expressed by their residuum [10]. The *QT* interval represents the ventricular depolarization and repolarization time interval. This interval is measured from the *Q* onset to the *T* end. As the *QT* interval decreases while the heart rate increases, the *QT* interval is usually normalized with respect to the heart rate, and it is denoted as *QT*c. The accuracy in the estimation of the *QT* interval depends on the accuracy in identification of *Q* onset and *T* end [11]. Accurate measurement of the *QT* interval could be difficult for some *T*-wave morphologies and noisy electrocardiogram signals [12]. Several research groups have shown that advanced high-resolution ECG (HRECG) processing enables the estimation of several advanced indexes related to cardiovascular diseases. The study of HRECG started in the seventies with the goal of noninvasive detection of electrical activity of the His–Purkinje system [13]. Later, other cardiac zones or ECG intervals included micropotentials related with pathological conduction of the heart [14].

Starc and Schlegel [15] have developed a system that enables real-time assessment of the *QT* interval variability in the eight independent leads of a standard electrocardiogram. This system includes hardware that performs the ECG signal acquisition at 1000 samples per second in each lead with a resolution of 12 bits per sample. The system also includes advanced HRECG software for estimating the score of reduced amplitude zones (RAZ) as well as heart rate variability (HRV) analysis either in time domain or in frequency domain. Among the advanced tools for ECG analysis, it includes the *T*-wave morphological multilead analysis based on principal component analysis (PCA). Based on this system, Schlegel et al. [16] have derived an advanced ECG score for prediction of coronary artery disease (CAD) which is based on a set of parameters estimated using the advanced software analysis. The software system known as Cardiosoft [17] runs on the windows operating system.

Karjalainen et al. [18] developed a method for quantifying the *QT* interval variability. The method is based on a technique known as principal component regression (PCR). This method allows reducing the dimensionality of data. The variation of the *QT* interval is proportional to the variation in a parameter representing the third eigenvalue of the correlation matrix. The algorithm divides the ECG signal into segments including the *QRS* and the *T* wave, and then, the PCR algorithm is applied. This algorithm allows studying the *QT* interval variability without detecting the *T* wave.

Christov and Simova [19] proposed an automated method that scored as second in the Physionet/CinC Challenge 2006. The method is based on analyzing the presence of peaks or slopes in two consecutive segments of 10 msec. The search is performed first for estimating the *Q* onset and then for the *T* end. A revision of several classical methods for *QT* detection is presented by Xue and Reddy [20].

The analysis of the ECG signal is becoming an important tool for the assessment of several cardiac diseases. In this context, we are proposing a system including hardware and software for HRECG analysis, where support vector machine (SVM) techniques [21–23] are used within the framework for *QT* interval estimation. The proposed system includes the signal acquisition electronics hardware as well as a software system for ECG signal analysis. The software system is based on open-source libraries and runs on the Linux operating system. The proposed system performs multilead *QT* interval estimation. The proposed methodology is based on the work reported by Starc and Schlegel [15] but we have used a different approach for preprocessing and for segmentation of the *QRS* template on the eight independent leads of the 12-lead standard surface electrocardiogram. In our algorithm, rather than relying on the *QRS* detector for determining the beginning of the *QRS* (*Q*_onset_) and a biparabolic curve modeling for determination at the end of the *T* wave (*T*_end_), we perform these tasks using SVM techniques. The SVM learning technique is a very useful tool for classifying ECG patterns [24]. This has motivated their application for accurate estimation of this interval. The *QT* interval estimation method is validated using the Physionet PTB diagnostic ECG database [25–27], as well as a dataset including a group of 60 patients acquired using our digital electrocardiograph system. The article is organized as follows: first, a brief introduction and state of the art is given, as well as a description of the theoretical bases, and then, the methodological framework is described. Subsequently, the results are presented, and finally, conclusions are given.

## 2. Methods

### 2.1. DIGICARDIAC ECG Acquisition System

The DIGICARDIAC system enables acquisition, processing, and handling of the electrocardiogram as well as the clinical patient information. The proposed system incorporates advanced algorithms for signal processing and interval estimation as well as telemedicine tools for handling the patient electronic record. The DIGICARDIAC instrument has two types of operation modes. First of all, it can be used as any electrocardiograph in a clinical consultation. Secondly, it works as an instrument of detailed analysis, normally used in cardiac clinical research.

**Figure 1:**
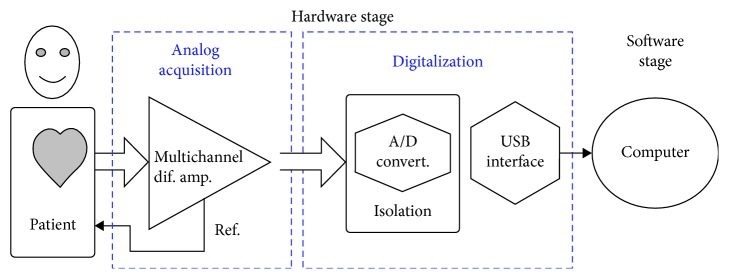
DIGICARDIAC system components.

**Figure 2:**
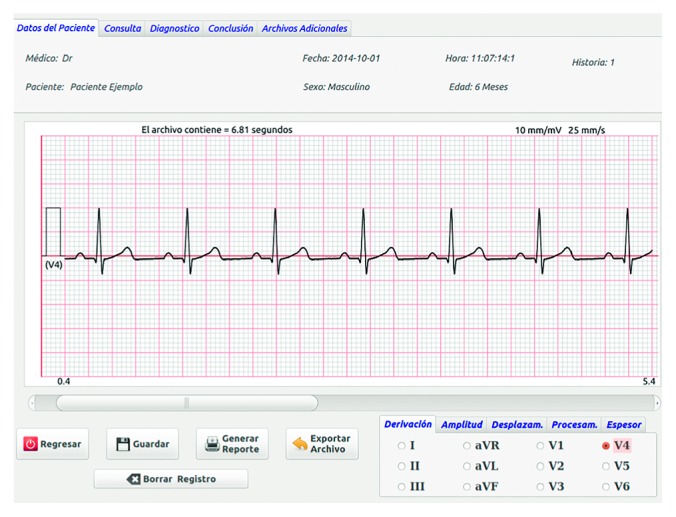
The DIGICARDIAC user interface window showing a graphical representation of the V4 ECG lead.

The electronic hardware of the DIGICARDIAC system [28] is used for acquiring the standard 12-lead electrocardiogram. This hardware system was designed to work as a whole, connected with a personal computer, as shown in Figure 1. The hardware starts working when it gets plugged into the USB port. The high-resolution electrocardiogram signals are transmitted constantly to the computer. The developed software tools running on the personal computer allow the user to initiate the electrocardiogram data recording; otherwise, the transmitted information from the hardware is discarded. The acquisition is performed using the standard 12-lead system at 2000 samples per second with a resolution of 12 bits per sample. An example of acquisition is shown in Figure 2, where the V4 recorded lead is shown.

#### 2.1.1. Acquisition Data

The protocol included three groups of subjects: 20 patients with Chagas disease, 20 patients with hypertension, and 20 healthy subjects. Patients underwent a high-resolution 12-lead electrocardiogram (ECG) recording using DIGICARDIAC. A standard supine 12-lead ECG was recorded for 5 minutes in each of the 60 patients during normal breathing.

### 2.2. Multilead *QT* Interval Measurement

The algorithm reported for estimation of the *QT* interval is multilead in the sense that the eight independent leads of the standard electrocardiogram are considered for estimating the *QT* interval. Particularly, the segmentation of the *QRS* template as well as the incoming beat involves stages where parameters are extracted from estimations performed on each of the leads. Additionally, even when an estimation of the *QT* interval is provided as a result for each of the leads, their combination allows one to provide a representative *QT* interval estimation for all leads. A description of the different processing stages is presented in the following paragraphs.

#### 2.2.1. Signal Preprocessing

The signal is preprocessed with a moving averaging filter for attenuating the power line interference. The baseline wandering was attenuated using a high-pass recursive filter and a FIR filter for attenuating the electromyographic noise, as reported in [19]. The phase characteristic is constant, and the filter was applied forward and then backwards for minimizing the phase distortion. The high-pass filter response is given by

(1) $$\begin{array}{c} y\left(n\right)=C_{1}\left(x\left(n\right)-x\left(n-1\right)\right)+C_{2}y\left(n-1\right), \end{array}$$

where *x*(*n*) is the input signal sequence and *y*(*n*) is the output filtered sequence, and coefficients *C*_1_ and *C*_2_ are calculated as follows:

(2) $$\begin{array}{c} C_{1}=\frac{1}{1+\tan\left(f_{\text{c}}\pi T\right)},\\ C_{2}=\frac{1-\tan\left(f_{\text{c}}\pi T\right)}{1+\tan\left(f_{\text{c}}\pi T\right)}, \end{array}$$

where *f*_c_ is a cutoff frequency of 0.64 Hz and *T* is sampling period. The next step is using the Savitzky and Golay filter [29] for attenuating the electromyographic noise. The filter is based on performing a least-square approximation of the signal given by

(3) $$\begin{array}{c} Y\left(n\right)=\frac{1}{M}\overset{N}{\underset{j=-N}{\sum }}C_{j}X\left(n+j\right), \end{array}$$

where *Y*(*n*) and *X*(*n*) are the signal after and before the least-square approximation, respectively, *N* is the length of the approximation interval at both sides of the sample, and *C*_*j*_ are the weighting approximation coefficients given by

(4) $$\begin{array}{c} C_{j}=3N^{2}+3N-1-5j^{2}, \end{array}$$

and the normalization coefficient *M* is given by

(5) $$\begin{array}{c} M=\frac{\left(2N+1\right)\left(4n^{2}+4N-3\right)}{3}. \end{array}$$

The procedure is applied for a total interval (2*N*+1) of 31 milliseconds. The next step during the preprocessing is the *QRS* detection, which is performed according to an adaptation of the Pan and Tompkins algorithm proposed by Mneimneh et al. [30], which uses a methodology based on thresholding. Then, a segmentation of the signal is made; this task consists in taking samples present in the ECG signal where the *QRS* complexes are present. Thus, the ECG record is divided into their component waves (a heartbeat is the representation of a cardiac cycle). The segmented ECG signals are used for constructing the *QRS* templates.

#### 2.2.2. Templates

**Figure 3:**
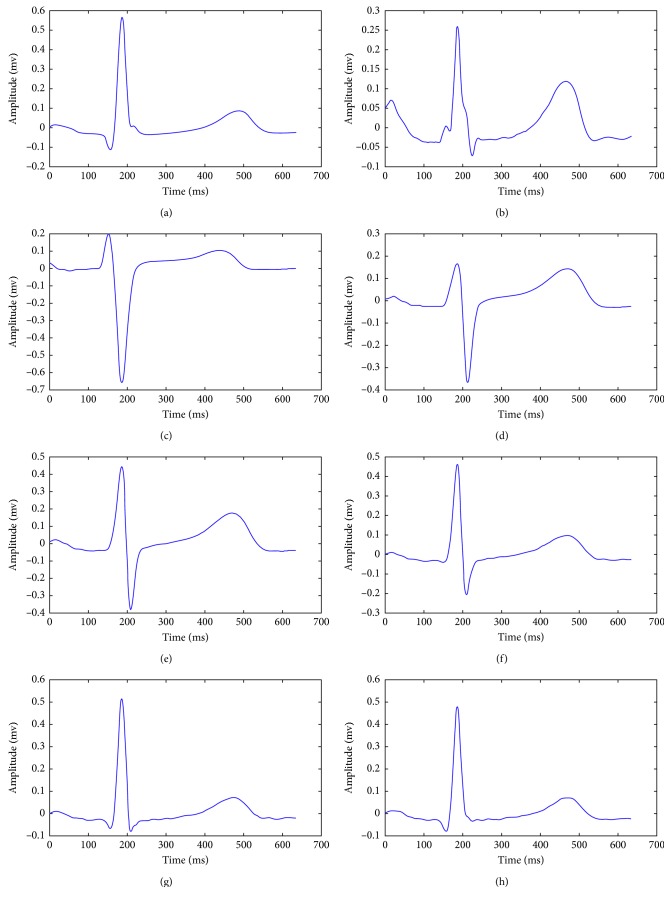
Templates for each of the 8 independent derivations of the ECG. (a) Deviation template I. (b) Deviation template II. (c) Deviation template V1. (d) Deviation template V2. (e) Deviation template V3. (f) Deviation template V4. (g) Deviation template V5. (h) Deviation template V6.

A histogram for the RR interval is constructed based on the first twenty beats extracted from the ECG signal. Then, an average *QRS* signal is obtained considering the ten beats with RR intervals closest to the maximum of the histogram. This averaged signal is a *QRS* template. The procedure is repeated for each of the eight independent leads of the standard electrocardiogram [15]. In Figure 3, the template for each independent lead is shown.

#### 2.2.3. *QRS* Template Segmentation

**Figure 4:**
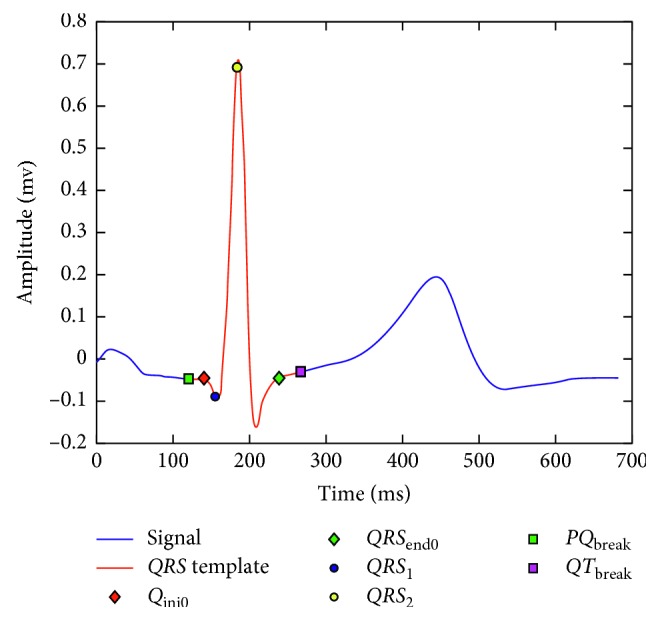
Template segmentation for obtaining *PQ*_break_ and *QT*_break_.

The fiducial point of the *QRS* is determined using the SVM technique in each lead template (this point is denoted as *Q*_ini_). The *QRS* detection algorithm provides the point *QRS*_1_ corresponding to the *QRS* wave onset, the point *QRS*_2_ corresponding to the *R* peak of the *QRS*, and the point *QRS*_end_ corresponding to the end of the *QRS*. A representative pair of values *QRS*_1_ and *QRS*_2_ for all leads is calculated using the procedure proposed by Starc and Schlegel [15] that enables calculation of the width of the *QRS* denoted as Δ*QRS*. The average *Q*_ini_ is obtained for the eight independent leads, and *Q*_ini0_ is selected as the value for the lead that occurs earliest with respect to the average. The point *QRS*_end0_ is selected using a similar procedure but considering the average for *QRS*_end_ points in the eight leads. The points *Q*_ini0_ and *QRS*_end0_ represent the initial and end points of the *QRS* wave. However, as suggested in [15], a larger interval is used by extending 20 ms before and 30 ms after the end, defining the points *PQ*_break_ and *QT*_break_, as shown in Figure 4.

#### 2.2.4. *T*-Wave Template Segmentation

**Figure 5:**
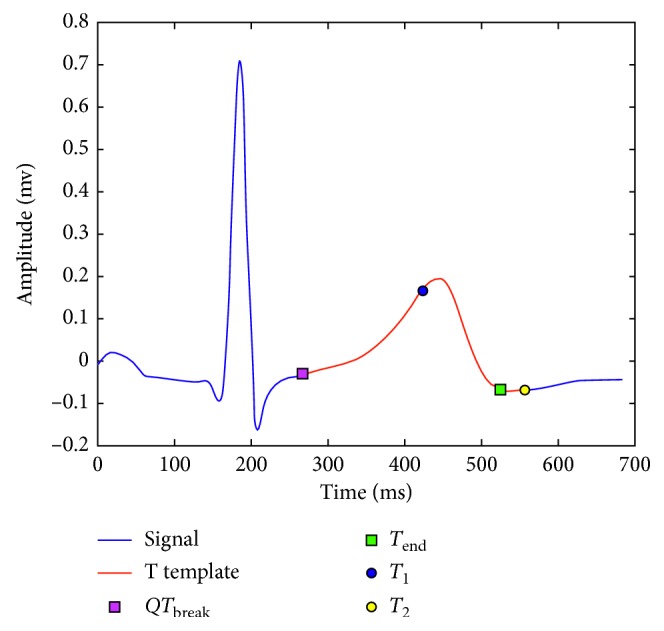
*T*-wave template segmentation between *QT*_break_ and *T*_2_.

The end of the *T* wave is estimated for each independent template lead using the SVM algorithm. This point is denoted as *T*_end_. The point *T*_2_ is located 30 ms after the *T*_end_ point, and *T*_1_ is located 20 ms before the maximum of the absolute value of the *T* wave. Location for the *T* wave on each template is defined between points *QT*_break_ and *T*_2_, as shown in Figure 5.

#### 2.2.5. Template *QT* Interval

The *QT* interval in each template lead is defined using the estimation for *Q*_ini0_ and *T*_end_. The calculation is performed as follows:

(6) $$\begin{array}{c} QT_{k,0}=T_{k,\text{end}}-Q_{k,\text{ini0}}, \end{array}$$

where *k* is the lead considered.

### 2.3. Beat-to-Beat *QT* Interval Estimation

After considering the first twenty beats necessary for template calculation and *QT* interval initialization, our algorithm performs the beat-to-beat *QT* interval calculation. Each incoming beat is compared with respect to the lead template and accepted when the Pearson correlation coefficient is larger than a predefined threshold. The accepted beat is segmented by obtaining *Q*_ini_ and *T*_end_ using SVM techniques, and then, the points *QRS*_1_, *QRS*_2_, *QRS*_end_, *T*_1_, *T*_2_, *PQ*_break_, and *QT*_break_ are obtained using a procedure similar to the template segmentation. The calculation of the *QT* interval for the incoming beat is performed by shifting the incoming signal with respect to the template to obtain the maximal correlation within a particular beat region. The amount of shifting is used for updating the *QT* interval on each lead using the procedure proposed by Starc and Schlegel [15]. The beat-to-beat *QT* interval estimation is calculated as follows:

(7) $$\begin{array}{c} QT_{k,i}=QT_{k,0}+\Delta QRS_{i}+\Delta T_{k,i}, \end{array}$$

where *i* is the incoming beat and *k* is the lead considered. The updating of the *QT* interval is the addition of the amount of shifting in the *QRS* template denoted as Δ*QRS*_*i*_ and the *T*-wave template denoted as Δ*T*_*k*,*i*_. The amount of shifting is calculated as explained in Section 2.3.3. The system provides as a result the sequence of *QT* intervals for each of the leads, as well as their average.

#### 2.3.1. Calculation of the Shifting for the *QRS* Template

**Figure 6:**
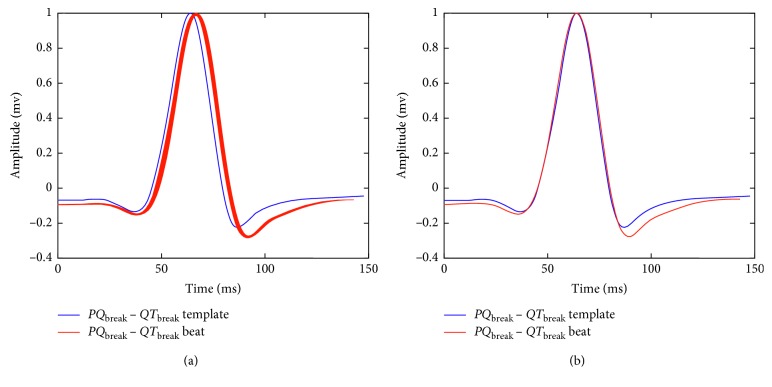
First stage for the alignment of the *QRS* template and the incoming beat. In (a), the initial alignment between the *QRS* template and the incoming beat is shown. In (b), both signals are aligned considering the procedure described in Section 2.3.3 for the interval between *PQ*_break_ and *QT*_break_.

**Figure 7:**
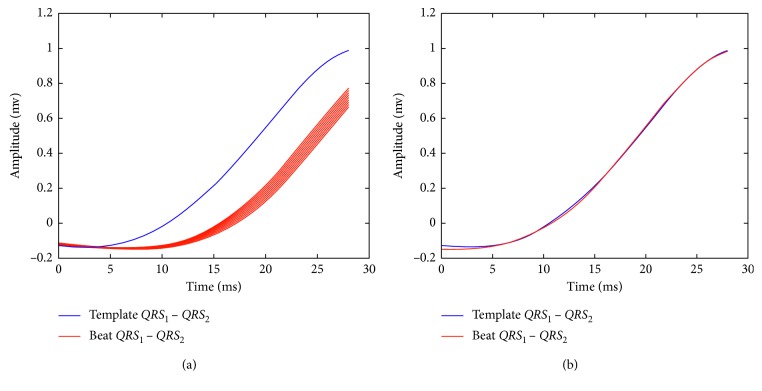
Second stage for the alignment of the *QRS* template and the incoming beat. In (a), the incoming beat is shifted with respect to the *QRS* template. In (b), both signals are aligned for the interval between *QRS*_1_ and *QRS*_2_.

The total amount of shifting for the *QRS* template is calculated considering two stages: in the first stage, the shifting of the segment *PQ*_break_ − *QT*_break_ in the incoming beat with respect to the similar interval in the *QRS* template is calculated. This process is illustrated in Figure 6. During the second stage, the amount of shifting of the segment *QRS*_1_ − *QRS*_2_ in the incoming beat with respect to the similar segment in the *QRS* template is calculated. The alignment for this segment is shown in Figure 7. This procedure is performed for each lead for obtaining the array Δ*QRS*_*k*,*i*_, where *k* denotes the lead and *i* is the incoming beat. From this array, a representative Δ*QRS*_*i*_ is calculated as described for the *QRS* template segmentation.

#### 2.3.2. Calculation of the Shifting for the *T*-Wave Template

**Figure 8:**
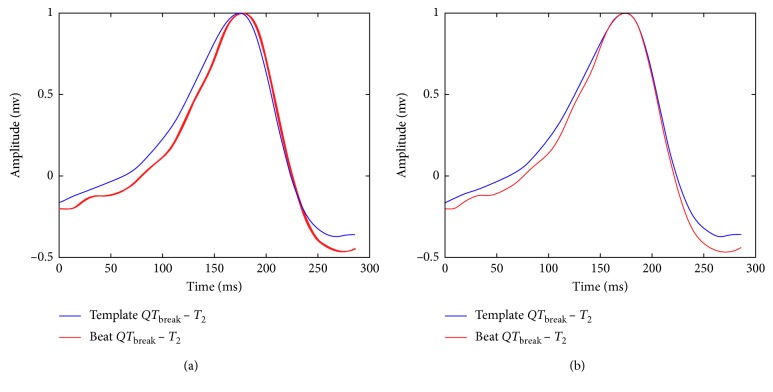
First stage for the alignment of the *T*-wave template and the incoming beat. In (a), the initial alignment between the *T*-wave template and the incoming beat is shown. In (b), both signals are aligned considering the procedure described in Section 2.3.3 for the interval between *QT*_break_ and *T*_2_.

**Figure 9:**
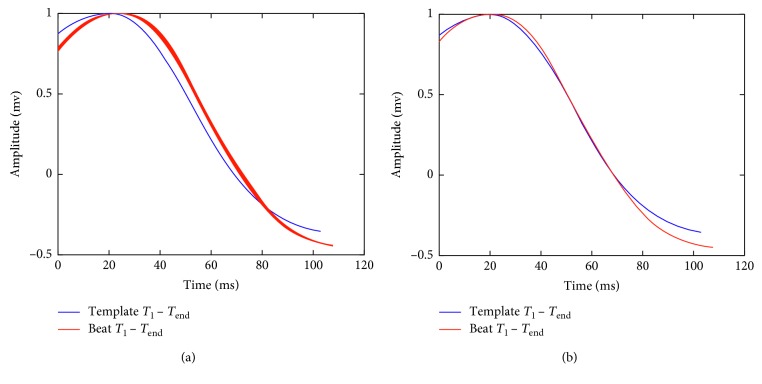
Second stage for the alignment of the *T*-wave template and the *T* wave in the incoming beat. In (a), the incoming *T* wave is shifted with respect to the *T*-wave template. In (b), both signals are aligned for the interval between *T*_1_ and *T*_end_.

The total amount of shifting for the *T*-wave template is also performed using two stages: in the first stage, the shifting is calculated for the segment between *QT*_break_ and *T*_2_ in the incoming beat with respect to the similar interval in the *T*-wave template. The alignment process is shown in Figure 8. During the second stage, the shifting is calculated for the *T* wave considering the segment between *T*_1_ and *T*_end_ in the segmented incoming beat with respect to the *T*-wave template. This alignment process is illustrated in Figure 9. This process is performed for each lead for obtaining the array of shifting for the *T* wave, Δ*T*_*k*,*i*_.

#### 2.3.3. Temporal Shifting between Two Signals

The amount of shifting between a template denoted as *x*(*n*) and the incoming signal denoted as *y*(*n*) is calculated using the following functional:

(8) $$\begin{array}{c} J\left(\theta \right)=\underset{\theta }{\text{min}}\overset{N}{\underset{n=1}{\sum }}{\left(x\left(n\right)-y\left(n\pm \theta \right)\right)}^{2}, \end{array}$$

where *θ* is the shifting in samples between signals and *N* is the length of the segment. The incoming signal is normalized with respect to the template.

#### 2.3.4. *Q*_ini_ and *T*_end_ Calculation Using SVM

Two support vector machine algorithms were trained for detecting these points. The first SVM is trained for detecting the *Q*_ini_ point, and the second SVM was trained for detecting the *T*_end_ point. The training for the first SVM is performed using as feature ECG signal intervals that are centered at the actual *Q*_ini_ location (defined by an expert). These intervals are called *markers*, and other ECG signal intervals selected from other ECG regions are called *nonmarkers*. The second SVM is trained similarly for detecting the *T*_end_ point.

*(1) Training*. A total of 1800 beats are used for extracting features for training. 600 beats are extracted from hypertensive patients, 600 beats are extracted from chagasic patients, and 600 beats are extracted from control subjects. An additional set of 300 beats (100 from each patient group) is used for validation.

The annotation of the actual location of *Q*_ini_ and *T*_end_ was performed manually on each beat by a group of cardiologists.

*(2) Features Used for the SVM*. The SVM training is done based on selected reference points called *markers* and *nonmarkers*. For each of the selected points, the features used are a vector including signal samples located around the selected point as well as signal samples extracted from the discrete wavelet decomposition [31] of the beat selected.

The choice of the mother wavelet is based on research studies about the application of different wavelet families for *QRS* complex and *T*-wave detection [32, 33]. These research works recommend the utilization of the wavelet family Daubechies-4 (level 5) for *QRS* complex detection and Symlet-7 (level 6) for the *T* wave, because these wavelets show coefficients with greater amplitude and a pattern that roughly identifies the starting and the end point of the *QRS* complex, as well as the start and end point of the *T* wave. In consequence, this type of wavelets is used in the present work.

**Figure 10:**
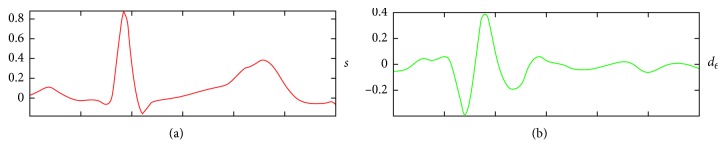
(a) A *QRS* signal template. (b) The IDWT reconstructed detail level 6 using Symlet-7. Two peaks are shown at the end of the *T* wave: one positive and one negative including a zero crossing.

**Figure 11:**
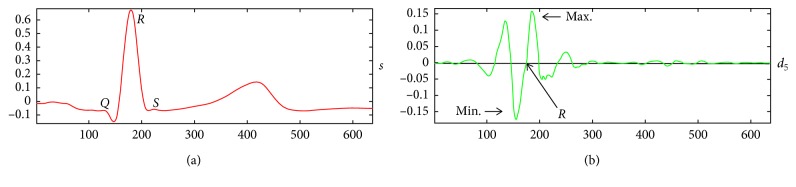
(a) A *QRS* signal template. (b) The reconstructed Daubechies-4 detail level 5. The zero crossings and peaks correlate with the *Q*, *R*, and *S* fiducial points.

In this case, a level 5 reconstruction of the Daubechies-4 wavelet is used for the *Q*_ini_ detection. A level 6 reconstruction using the Symlet-7 is used for the *T*_end_ detection. The level of detail d6 offers relevant information about the *T* wave, by characterizing this pattern with two peaks: one positive and one negative including a zero crossing (Figure 10). Both reconstructed signals are obtained with the same size as the *QRS* template. In Figure 11, the time matching between the original *QRS* signal and its wavelet reconstruction is shown. In this case, the level 5 detail of the Daubechies-4 wavelet decomposition is reconstructed using the inverse discrete wavelet transform (IDWT), where the zero crossings and peaks correlate with the *Q*, *R*, and *S* fiducial points.

**Figure 12:**
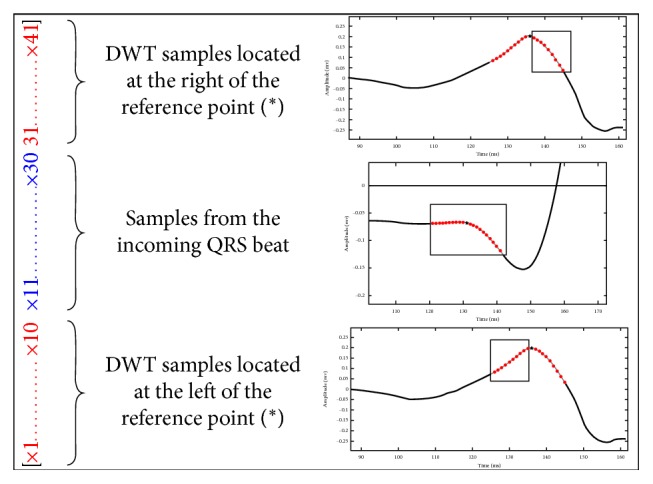
Feature vector for training the SVM used for detecting the *Q*_ini_.

*(3) Features Vector*. Each feature vector for *markers* and *nonmarkers* points is a vector including 41 samples, where samples 1 to 10 are the samples of the reconstructed wavelet detail located at the left of the selected *Q*_ini_ fiducial point. Samples 11 to 30 are the samples in the ECG beat located on a window centered on the selected point, and samples 31 to 41 are the samples of the reconstructed wavelet detail located at right of the selected *Q*_ini_ fiducial point. The construction of each marker vector is illustrated in Figure 12. At the left of the figure, the features vector content is shown. At the right, the window of the data source used for constructing the features vector is shown. The top and bottom are the reconstructed wavelet details of the beat, and at the center is the data from the corresponding *QRS* beat. The markers for the *T*_end_ are constructed similarly; however, the wavelet decomposition uses a Symlet-7 wavelet, and the reconstructed detail 6 is considered for representing the marker. The length of the marker in this case is longer (51 samples) but the difference is that samples from 11 to 40 are extracted from the incoming beat. The rest of samples comes from the reconstructed wavelet detail.

*(4) Nonmarker Selection*. The *nonmarker* features vector is constructed with the same size and composition as the *marker* feature vector. However, in this case, the samples from the ECG beat and reconstructed wavelet detail are located outside from the marker regions. The location is selected at random from the beat and far from the fiducial point (*Q*_ini_ or *T*_end_). For each *marker* vector, a number of five *nonmarker* vectors are selected. This procedure was performed for each of the 1800 beats considered for training.

*(5) Parameters Tuning*. Parameters gamma and sigma of the SVM were set using the following procedure: the detection of the fiducial point was performed in incoming beats extracted from the test set for classification accuracy estimation. The parameters were varied for obtaining a classification accuracy larger than 95%. The selected parameters for the *Q*_ini_ were gamma=900 and sigma=25 and for the *T*_end_ were gamma=10 and sigma=60.

*(6) Detection of *Q*_ini_ and *T*_end_ Using the Trained SVM*. The detection of *Q*_ini_ and *T*_end_ points is performed using the following procedure:

1. In each incoming beat, the level 5 reconstruction using the IDWT is obtained using the db4 wavelet. Similarly, the level 6 reconstruction using the Symlet-7 wavelet is calculated.
2. A search window is established, taking as reference the fiducial points calculated for the template on each lead: *Q*_*k*,ini0_ and *T*_*k*,end_. Thus, the search window for *Q*_ini_ is centered in the approximated initial fiducial point for the current lead, and it has a length of seven samples in the interval [*Q*_*k*,ini0_ − 3, *Q*_*k*,ini0_, *Q*_*k*,ini0_+3]. Similarly, for the end of the *T* wave, the search window is located in the following interval: [*T*_*k*,*end*_ − 3, *T*_*k*,*end*_, *T*_*k*,*end*_+3].
3. For each of the points in the search window, the features vector is constructed and classified using the SVM. The points classified as *markers* are labeled with −1 and *nonmarker* with +1.
4. The points with a predicted label of −1 are selected. If there is more than one point with a predicted label −1, the first point of the group is selected as *Q*_*k*,ini_. In the event that no point is labeled with −1 during the *Q*_ini_ detection, the parameter is set as *Q*_*k*,ini_=*Q*_*k*,ini0_ using the value calculated for the initial template. Concerning the *T* wave, when there is more than one point with predicted label −1, the last point of the group is selected as *T*_*k*,end_. Similarly, in the event of no points labeled with −1, the parameter is set as *T*_*k*,end_=*T*_*k*,end0_ using the value calculated for the initial template.

**Figure 13:**
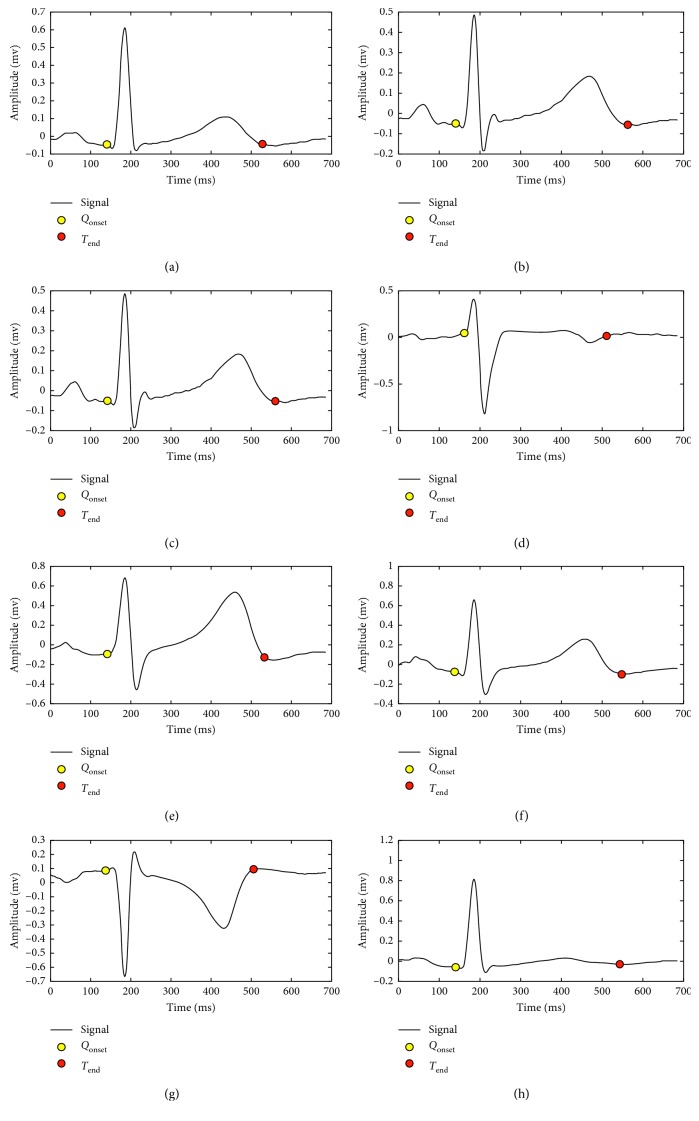
Detection of *Q*_onset_ and *T*_end_ points for different morphologies of the heartbeat.

Figure 13 shows a set of beats with different morphologies and its corresponding *Q*_ini_ and *T*_end_ points detected with the SVM.

## 3. Results and Validation of the Algorithm

### 3.1. Validation Using Synthetic Signals

A test using simulated signals was performed based on the work reported in [34]. A heartbeat was selected, and the amplitude of the *T* wave was varied by reducing their amplitude to 80%, 50%, and 30%. This *QRS* was replicated to obtain four signals with 300 beats, and white noise was added with zero mean and standard deviation of 3% of the original *T*-wave amplitude. Each signal was replicated to complete the eight independent leads. The set of signals have a constant *QT* of 391 msec; in consequence, the expected *QT* variability is 0 msec. The estimated *QT* for this experiment attained a standard deviation of 1.4 msec.

### 3.2. Validation Using the Physionet Database

The multilead *QT* estimation method is validated using a subset of the PTB Diagnostic ECG annotated database [25, 35]. This subset includes a group of 97 subjects where 80 belong to the control group, 7 are patients with hypertrophy, 6 are patients with valvular disease, and 4 are patients with myocarditis. The database contains the standard 12-lead ECG and the simultaneously recorded 3 Frank lead ECG. The signals are sampled at 1000 Hz, with a resolution of 0.5 *μ*V, and they have variable duration.

The validation process was performed by comparing the values of the *QT* interval calculated by our algorithm with respect to the manually estimated values of the *QT* parameter obtained by a group of Physionet experts (this information is provided in a text file for this dataset). The comparison is expressed in terms of the percent error; the percentage error obtained for each patient is reported as well as the mean and standard deviation of each of these measurements. Results of the validation using the Physionet PTB diagnostic ECG dataset are shown in the first row of Table 1. The first column corresponds to the group analyzed, the second column corresponds to the value (mean ± std (min, max)) of the *QT* parameter in the Physionet reference file (*QT* phy.), the third column represents the value of *QT* calculated with our *QT* interval estimation algorithm (QT est.), and the last column shows the percentage error for *QT* interval (error (%)).

The annotated *QT* interval considering the 97 patients of the Physionet database is 387.83 ± 32.57 msec with a minimum of 317.00 msec and a maximum of 477 msec. The estimated *QT* interval is 384.74 ± 32.61 msec, with a minimum of 316.90 msec and a maximum of 474.60 msec. The average percentage error is less than 5%, 2.60 ± 2.25. The results obtained with this subset of the Physionet database are close to results reported by participants of the 2006 *QT* estimation challenge as Christov and Simova [19]. However, our approach is multilead and intended for *QT* variability analysis.

The second row of Table 1 reports the values corresponding to the *QT* interval obtained when processing the 80 electrocardiography records of control subjects using our algorithm compared with respect to the values provided in the Physionet reference file. The calculation of the interval *QT* indicates a good correlation with respect to the estimation provided in the Physionet dataset, presenting an average error of 2.35%. This set of control subjects belongs to the test set, and the percent of error is low demonstrating the good performance of the algorithm proposed. The third row of Table 1 reports the results obtained for the *QT* interval estimation, considering the 7 electrocardiography records of patients with myocardial hypertrophy. The results are compared with respect to the values provided in the Physionet reference file. In this case, the percentage of error for the mean *QT* interval is less than 5%, presenting a maximum error of 8.2% and a minimum error of 1.42%.

Results of *QT* intervals obtained when processing the 6 electrocardiographic records of patients with valvular heart disease using our algorithm with respect to the values provided in the Physionet reference file are presented in the fourth row of Table 1. The automatic estimation obtained by the algorithm provides good results, with an average error for the parameter *QT* less than 4%.

The fifth row of Table 1 reports the estimated *QT* interval values obtained when processing the 4 electrocardiography records of patients with myocarditis with respect to the values contained in the Physionet reference file. The values for the average percent error are 3.11%, and the standard deviation is 2.48%.

The results shown in Table 1 are satisfactory since this validation group (test data) is not part of the SVM training dataset and even though the average percent error for the mean and standard deviation is lower than 5%. These results show the good performance of the algorithm proposed in this work. Similarly, it is worth to mention there is a good correlation with respect to the values obtained for the *QT* interval reported in [36] and using the same Physionet dataset [26]. Results in Table 1 show globally an underestimation with respect to manual annotations of the Physionet dataset of 3.09 msec. Such underestimation could be explained by the fact that annotators performed the manual estimation of the *QT* interval using only the lead II for the Physionet dataset [35] used in this validation. In contrast, our algorithm performs the *QT* estimation using a multilead approach, where all leads have a similar weight as we are simply averaging the *QT* intervals measured in each of the leads. A bias in the estimation using this dataset has also been reported by other researchers [37, 38] during the validation of multilead *QT* estimation algorithms.

### 3.3. Validation with a Dataset Acquired Using DIGICARDIAC

Results of the validation using 60 patients acquired with the DIGICARDIAC system are shown in Table 2. The validation for this dataset is performed by comparing the average *QT* interval in the eight leads with respect to the *QT* interval calculated using the Cardiosoft software [17]. The average *QT* interval estimated by the Cardiosoft software is 389.53 ± 30.37 msec with a minimum of 320.00 msec and a maximum of 450.00 msec. The average *QT* interval estimated using our algorithm is 391.62 ± 30.94 msec with a minimum of 318.40 msec and a maximum of 453.30 msec. The mean and standard deviations of the *QT* differences between our method and the Cardiosoft estimation are 2.49 ± 1.99 msec. The minimum is 0.08 msec, and the maximum is 8.23 msec.

Table 3 shows the numerical results corresponding to the parameter *QT* (msec) and *QT*c (msec), obtained by processing 20 electrocardiography records of hypertensive patients using our algorithm and the CardioSoft application. The average percentage error for *QT* is 1.74%, with a minimum value of 0.07% and a maximum value of 4.38%. Regarding the *QT*c, the average error of 2.66% is obtained with a minimum of 0.07% and a maximum of 5.20%. In this case, the error is below 6%, which is an acceptable measure.

The numerical results corresponding to the *QT* interval (msec) and *QT*c (msec) obtained by processing 20 electrocardiographic records of chagasic patients using the algorithm proposed and the CardioSoft application are shown in Table 4. The results provided by the proposed algorithm show a good accuracy as the average error for the parameters *QT* and *QT*c is less than 4%.

Table 5 shows the numerical results corresponding to the *QT* and *QT*c parameter in msec. In this case, the estimation is performed for 20 electrocardiography records of control subjects (healthy) using the proposed algorithm and compared with respect to the CardioSoft application. Concerning the *QT* interval, the mean value of the error was 2.54%, which is an acceptable measure. Regarding the *QT*c, the average error is 1.87%. These low error values show that the algorithm proposed could be useful for clinical application research.

The results shown in Tables 2–5 reflect the good performance of the methodology proposed in this research. The algorithm offers a good accuracy for the estimation of the *QT* and *QT*c intervals with respect to the estimated values obtained using the commercial software provided by CardioSoft. The comparison shows an average error of less than 4%.

## 4. Statistical Comparison between Patients and Control Subjects

In this section, we report the results of using one-way analysis of variance to determine statistically significant changes in the *QT* and *QT*c parameters between groups of patients. One-way analysis of variance was used to determine statistically significant changes (*p* < 0.05 and *F* > 1) between groups of patients. The use of this type of statistical analysis is justified because there are independent samples of two groups, and we want to contrast the null hypothesis (equality of means) with the hypothesis that the means are not equal.

Table 6 summarizes the results obtained for the *QT* and *QT*c parameter between hypertensive patients and control subjects in mean ± SD.

As can be seen, the average *QT*c and *QT* intervals are larger for the group of hypertensive patients in comparison with the control group. It is also important to note that there are statistically significant differences between hypertensive patients and control subjects represented by a value of *p* < 0.05. In general, the *QT* and *QT*c interval is prolonged in hypertensive patients with respect to healthy subjects as reported in [39–41].

Results obtained for the comparison of chagasic patients and healthy subjects concerning the *QT* and *QT*c interval are reported in Table 7. Results show statistically significant differences for *QT* and *QT*c intervals between control subjects and chagasic patients (*p* < 0.05). Concerning this disease, further research is still necessary considering a larger group of patients and handling exclusion criteria concerning other diseases affecting the left ventricular repolarization [42].

## 5. Conclusions

The DIGICARDIAC system incorporates a software tool that enables accurate estimation of the multilead *QT* interval variability. The *QT* interval estimation algorithm uses high-performance machine learning techniques such as support vector machines for accurate estimation of fiducial points *Q*_ini_ and *T*_end_ on a multilead *QT* estimation framework. The algorithm was validated using the Physionet *QT* interval dataset, and additionally, a second validation was performed with respect to other commercially available software tools such as CardioSoft. Results obtained during the validation process are promising as the mean percentage error and deviation standard are low. The validation performed on hypertensive patients compared with respect to healthy subjects confirms the presence of *QT* interval prolongation in HTA patients with respect to the normal subjects. In contrast, the validation considering Chagas disease patients suggests the need of improving the clinical protocol considering a more strict selection of patients.

## Figures and Tables

**Table 1:** Validation of *QT* estimation using the Physionet database. The parameter is represented as mean ± std. (min., max.).

Group	*QT* phy. (msec)	*QT* est. (msec)	Error (%)
Physionet dataset	387.83 ± 32.57 (317, 477)	384.74 ± 32.61 (316.9, 474.6)	2.60 ± 2.25 (0, 14.11)
Control subjects	386.96 ± 32.66 (317, 477)	384.64 ± 32.36 (316.9, 474.6)	2.35 ± 1.85 (0, 9.22)
Myocardial hypertrophy	380.14 ± 39.36 (317, 426)	375.64 ± 43.98 (321.3, 438.1)	4.36 ± 2.17 (1.42, 8.2)
Valvular heart disease	394.58 ± 30.79 (346, 426)	383.86 ± 32.67 (344.3, 438.1)	3.64 ± 5.20 (0.49, 14.11)
Myocarditis	408.37 ± 18.79 (385.5, 427.0)	403.85 ± 10.17 (395.1, 418.5)	3.11 ± 2.48 (0.27, 5.92)

**Table 2:** Validation considering 60 patients acquired with DIGICARDIAC. The validation is performed by comparing with respect to the Cardiosoft estimation.

	*QT* Cardiosoft (msec)	*QT* est. (msec)	Error (%)
Mean	389.53	391.62	2.49
Std. dev.	30.37	30.94	1.99
Min.	320.00	318.40	0.08
Max.	450.00	453.30	8.23

**Table 3:** *QT* interval (msec) and corrected *QT* (msec) for hypertensive patients with respect to the Cardiosoft application.

Parameter	*QT* est.	*QT* card.	*QT E*_r_ (%)	*QT*c est.	*QT*c card.	*QT*c *E*_r_ (%)
Mean	389.92	391.2	1.74	421.31	423.95	2.66
Std. dev.	26.63	29.63	1.36	21.58	20.49	1.33
Min.	348.8	348	0.07	379.7	380	0.07
Max.	447.6	450	4.38	452	464	5.20

**Table 4:** *QT* interval (msec) and corrected *QT*c (msec) for chagasic patients compared with respect to the Cardiosoft application.

Parameter	*QT* est.	*QT* card.	*QT E*_r_ (%)	*QT*c est.	*QT*c card.	*QT*c *E*_r_ (%)
Mean	400.05	395.1	3.20	423.29	414.8	3.38
Std. dev.	36.30	45.34	36.30	21.30	24.85	2.68
Min.	318.4	300	0.17	378.4	376	0.09
Max.	453.3	494	8.23	476.6	466	9.34

**Table 5:** *QT* interval (msec) and corrected *QT*c (msec) for healthy subjects with respect to the Cardiosoft application.

Parameter	*QT* est.	*QT* card.	*QT E*_r_ (%)	*QT*c est.	*QT*c card.	*QT*c *E*_r_ (%)
Mean	384.88	376.6	2.54	434.74	428.45	1.87
Std. dev.	28.64	27.95	2.06	25.97	21.76	1.76
Min.	332	320	0.20	392.8	387	0.04
Max.	434.2	420	6.5	492.4	466	6.05

**Table 6:** Comparison for the *QT* and *QT*c interval for hypertensive patients and control subjects.

Parameter	Hypertensive (mean ± std. dev.)	Healthy (mean ± std. dev.)	*p* < 0.05
*QT* (msec)	389.29 ± 27.06	383.56 ± 31.18	0
*QT*c (msec)	422.51 ± 25.31	434.56 ± 29.11	0

**Table 7:** Comparison for the *QT* and *QT*c interval variability for chagasic patients with respect to control subjects.

Parameter	Chagasic (median ± SD)	Healthy (median ± SD)	*p* < 0.05
*QT* (msec)	398.90 ± 35.0	383.56 ± 31.18	0
*QT*c (msec)	420.60 ± 27.01	434.56 ± 29.11	0

## Data Availability

This research used a third-party dataset available online for validation purposes. The dataset is the PTB Diagnostic ECG Database available at https://physionet.org/physiobank/database/ptbdb/.
